# Auditory Hallucinations in Acute Stroke

**DOI:** 10.1155/2005/641953

**Published:** 2006-02-27

**Authors:** Yair Lampl, Mordechai Lorberboym, Ronit Gilad, Mona Boaz, Menachem Sadeh

**Affiliations:** ^1^Department of NeurologyEdith Wolfson Medical CenterHolonSackler Faculty of MedicineTel Aviv UniversityIsrael; ^2^Department of Nuclear MedicineEdith Wolfson Medical CenterHolonSackler Faculty of MedicineTel Aviv UniversityIsrael; ^3^Epidemiology Unit and the Institute for Cardiovascular ResearchEdith Wolfson Medical CenterHolonSackler Faculty of MedicineTel Aviv UniversityIsrael

## Abstract

Auditory hallucinations are uncommon phenomena which can be directly caused by acute stroke, mostly described after lesions of the brain stem, very rarely reported after cortical strokes. The purpose of this study is to determine the frequency of this phenomenon. In a cross sectional study, 641 stroke patients were followed in the period between 1996–2000. Each patient underwent comprehensive investigation and follow-up. Four patients were found to have post cortical stroke auditory hallucinations. All of them occurred after an ischemic lesion of the right temporal lobe. After no more than four months, all patients were symptom-free and without therapy. The fact the auditory hallucinations may be of cortical origin must be taken into consideration in the treatment of stroke patients. The phenomenon may be completely reversible after a couple of months.

## 1. Introduction

Auditory hallucinations are defined as auditory experiences without the presence of external stimuli. Descriptions of hallucinatory displays as being similar to deficit phenomena after stroke have been documented [1,3,4,17]. Auditory hallucinations are less commonly seen complications after stroke, mostly described as happening after ischemic or hemorrhagic lesions in the brainstem [13,21,25], the thalamus [11, 26] and the left internal capsule [18]. Auditory hallucinations after cortical strokes are very uncommon, and only few cases were reported describing this phenomenon. Anegawa et al. [1] reported unilateral auditory hallucinations after left temporal lobe ischemia. Augustine et al. [2] reported musical hallucinations following stroke of the right middle cerebral artery.

We analyzed in a prospective study all acute stroke patients who were admitted to our neurology department during the last six years and developed post stroke auditory hallucinations.

## 2. Patients and methods

Of the 641 patients (401 male and 240 female), who were admitted to our neurology department under the diagnosis of stroke during the years of 1996–2001 for primary investigation, 521 fulfilled the inclusion criteria and were included in the study. The mean age was 73.2 $\pm$ 12.0 years. The inclusion criteria for auditory hallucination of organic origin were as follows: the existence of true non-affective hallucination with the inability to maintain any control, distress or copying mechanism, known neurological origin, absence of psychiatric disorder and insight into the unreality of any pathological perspective state. Auditory hallucinations were defined as post acute stroke hallucinations if they appeared between the onset of symptoms and 14 days after the onset of stroke. Exclusion criteria included those patients with evidence of an additional disorder of the central nervous system apart from ischemic stroke, including inflammatory diseases or infections of the CNS, tumors, and

ISSN 0953-4180/05/$17.00 © 2005 – IOS Press and the authors. All rights reserved neurodegenerative diseases of the brain. Excluded were also patients taking recreational drugs or alcohol abuse patients, as well as those taking medications which had been proven to evoke hallucinations, including beta blockers or antihypertensive medications. Patients with past psychiatric history of affective or non-affective psychoses were excluded as well. Each patient underwent an acute stroke work-up which included repeat CT scans, carotid duplex examinations, transthorax and transesophogastric heart echo and comprehensive blood examinations. Each patient who demonstrated signs of a hallucinatory disorder underwent a protocol which included interviews with a neurologist and a psychiatrist. The questions were mainly directed at obtaining an exact description of the hallucination, using the Hamilton Depression Scale [14] and the Minimental State Examination (MMSE) [12]. The patients with hallucinations underwent additional testing with MRI scans and repeat electorencephalography (EEG) during the ictal phase, as well as during the interictal phase of the hallucinations. Repeat examinations, including ENT testing, as well as audiometry and brainstem evaluated potential test (BEAR) for the auditory hallucinations were carried out as well. The data from these examinations were compared with a 40 member aged- and sex-matched post cortical stroke patient group (mean age 63.2 $\pm$ 9.7 years) without hallucinations. The comparison was performed according to the anatomical characteristics of each stroke. Each stroke was diagnosed according to the anatomical location and volume [7].

## 3. Results

521 patients (340 male and 181 female) were primary included in the study. All were interviewed for having hallucinations. 16 (3%) patients had at least one kind of hallucinations (visual, auditory or tactile). Of these, 4 (2 male and 2 female) had auditory hallucinations. All cases of hallucinations occurred after cortical stroke. No patient demonstrated post stroke hallucinations after an infarction of the brain stem or subcortical structures.

In all four patients with auditory hallucinations, the hallucinations had the same characteristics which consisted of two main features: (1) hearing familiar persons, mostly family members speaking to the patients or between themselves or (2) hearing themselves in their own voice speaking. The data of patients is shown in Table 1.

## 4. Case descriptions

### *4.1. Patient No. 1*

A 67-year-old right handed woman was admitted to the hospital due to sudden onset of headache, dizziness and auditory hallucinations which appeared two days prior to hospitalization. She kept complaining of hearing her sister and neighbor continuously speaking between them and was convinced that she, herself, was saying things, events that other members of the family denied the presence of any speech by the patient. She had long-standing history of good health and no previous psychiatric event.

On admission, the patient was alert, well orientated as to time and place and had no speech or language difficulties. On the left side, mild hemiparesis and hemihypoesthesia was found. The deep tendon reflexes were brisker on the same side and accompanied by pyramidal signs.

In the next three weeks, the patient demonstrated continued vivid and clear auditory hallucinations. The hallucinations were complex, consisting of three types: (1) family members and neighbors speaking to her – the content of the speech was about sharing information regarding family members, including past information about a pet dog, having no threatening or frightening components and was not emanating from an external source; (2) the family members speaking with other persons, not about her, but projecting her ideas; and (3) hearing herself speaking in her own voice. She defined the last two incidents as unpleasant and threatening. The patient always declared that the spoken information came only from the right side.

Mental status tests were normal, including Waschler Adult test (WAT) (91 percentile) (27), Mini Mental State Examination (MMSE) (29/30) [12], digit span test, drawing a clock [24] and Trail Making Test [23]. Audiometric and repeat psychiatric examinations were normal. Electroencephalo-graphical examination revealed normal background with persistent appearance of waves in the right mid-temporal region without epileptic discharge. On the BEAR test, symmetrical BTT values $<0.2$ mg were found. On repeat CT scan and MRI, an acute lesion of the right temporal area was demonstrated. The infarction involved the right temporal auditory region (Broadman area 41, 42) (Fig. 1) and the post auditory area (interictal) (Broadman 22). SPECT HMPAO imaging demonstrated a hypoperfusion located in the middle and the posterior temporal lobe of the right side. The patient

Table 1

| No. | Age | Sex | Handedness | Neurological symptoms and/or complaints | Location of stroke | Treatment symptoms | Period of risk factors | Vascular |
|---|---|---|---|---|---|---|---|---|
| 1 | 67 | F | Right | Hemiparesis, dizziness, headache | Right temporal lobe | Haloperidol | 4 months | – |
| 2 | 56 | F | Right | Headache | Right temporal Lobe, right occipital lobe (old lesion) | Haloperidol and Carbamazepine | 1 months | Hypertension, diabetes mellitus |
| 3 | 72 | F | Left | – | Right temporal lobe | Haloperidol and Carbamazepine | 2 months | Hypertension, hyperlipidemia |
| 4 | 32 | M | Right | Left hemiparesis, left hemihypoesthesia | Right insula, right temporal lobe | Resperidone Carbamazepine | 3 months | Smoking |

was treated with haloperidol with good effects. Four months later, the medication could be discontinued successfully.

### *4.2. Patient No. 2*

A 56-year-old right handed woman with well-controlled hypertension and diabetes mellitus, was reported as having an episode of syncope, sudden headache and auditory hallucination following the syncope. A family member noted the abnormal event, but the patient refused to leave the house for medical attention. The family members were confused by her complaints of "telling them all what she thought" without actually hearing any of her words. She told them also that she heard both her sisters speaking the whole time between them, leaving her out, without their being physically present in the room. On neurological examination, no deficit was found. After admission, she continued to describe two types of hallucinations: (1) hearing herself in her own voice speaking in a well constructed manner; and (2) hearing her near relations discussing things above her head. The hallucinations consisted of very frightening characters. She did not define her condition as anxious, but as bizarre and unpleasant. The hallucinations occurred when she was not awake, and the voice was not of an external source.

On repeat psychiatric examination, no abnormality was found. Neurocognitive tests, including WAT (93 percentile), MMSE test (30/30), drawing a clock and trail making test, were all normal. Audiometric and evoked potential tests were within normal limits. During an episode of hallucination and interactive period, EEG examination showed no epileptic discharges with intermittent spikes in the mid and posterior right temporal region on a normal background. The BEAR examination showed a difference in the BTT of 0.3 msec between the right and left ear.

CT scan demonstrated two hypodense areas – an acute lesion of the right temporal post auditory region and a fixed lesion of the lateral periventricular region of the occipital lobe (Fig. 2). The patient was successfully treated with haloperidol combined with carbamazepine. The medications were discontinued one month after the onset of treatment.

### *4.3. Patient No. 3*

A 72-year-old left handed woman, suffering from hypertension, and hyperlipidemia, was examined by a psychiatrist after sudden onset of auditory hallucinations. According to her husband, she was in good health when she went to sleep, and during the night awoke, complaining of headache and saying that she heard her mother speaking in the other room. She was aware of the fact that her mother had died several years ago and that this phenomenon was illogical; and therefore, became very frightened and confused. One day later, the patient complained of hearing herself telling what she has to do and discussing problems about which she had no insight. She has a suspicion that the voice did not come from an external source, but was not sure. The commands consisted of short phrases of 2–3 words and were constructed well grammatically.

In the emergency room, no abnormalities were found on the neurological examination. The psychiatric examination revealed no pathology, apart form the auditory hallucination and severe anxiety state. Audiometric and evoked potential tests were normal. On EEG examination, slowing of the background in the tetra wave rate (more on the right side), but without epileptic discharge, was found during and after the hallucination period. BEAR examination was symmetrical with BTT less than 0.2 msec. On repeat brain CT scan, acute ischemic infarct of the middle upper region of the right temporal lobe was found.

She started a combined haloperidol and carbamazepine therapy which had very good efficacy. After one week, the auditory hallucinations disappeared, and the anti-psychotic treatment was discontinued. A trial of discontinuing the anti-convulsive medication two months later was successful.

### *4.4. Patient No. 4*

A healthy 32-year-old right-handed man, who was a smoker and had no vascular risk factors, was admitted to the hospital after the appearance of weakness of the left side combined with auditory hallucinations. He awoke one morning hearing himself speak that he feels bad. He called to his wife who immediately called an emergency care ambulance. During the transport to the hospital, he was very anxious, irritated and told the attendants for the entire transport time that he heard his wife and his children speaking to him about his condition. The neurological examination revealed moderate left hemiparesis with hemihypoesthesia.

On the following days, the auditory hallucinations became more intense and were concentrated in two main topics: (1) hearing himself speak about his condition without any real verbal speech and (2) hearing his wife and older daughter discuss with him about non-medical issues. The messages were very specific and very well constructed. He was only partially aware of the unreality of the hallucinations, plus very irritated, nervous and apprehensive. He has the feeling that the information came from an internal source, but also from an external one. CT scan showed involvement of the right insula and right superior temporal gyrus and part of the surrounding area 22.

Psychiatric examination did not reveal any abnormality, apart from the hallucinations. Neurocognitive tests (WAT – 94 percentile, MMSE – 30/30) were normal. Audiometric and evoked potentials, as well as repeat EEG examinations which showed a normal background with intermittent slowing on the right anterio-temporal area, were all normal. BEAR BTT was symmetrical at 0.25 msec. On brain CT scan, infarction of the right insula and right superior temporal gyrus and the surrounding Broadman area 22 was found (Fig. 3).

Two weeks after admission, the hallucination symptoms disappeared. The patient was transferred to a rehabilitation center where he stayed for the next five weeks. A combination therapy of resperidone-carbamazepine was continued for the next three months. The patient continued to act normally, having only moderate motor impairment. Unfortunately, one year later he was admitted again to the hospital with a recurrent stroke of the contralateral temporal lobe expressed as a central bilateral deafness.

## 5. Discussion

Auditory hallucinations have been rarely described after stroke, mostly after brain stem infarction [13,25]. Hallucinations of the non-peduncular type after cortical lesions are also supposed to be very uncommon. Most of the studies stress an association between the location of the cortical stroke in the dominant hemisphere and the appearance of auditory hallucination [15]. In the presented cases, all infarctions were localized on the right side of the brain. In three of the patients, the right side location was the non-dominant hemisphere. Whereas, the fourth patient was a 72-year-old female with characteristics of left-handedness on examination, but suspicion of a history of attempts to change hand dominance during early childhood. This raises the possibility that auditory hallucinations are associated with cortex lesions of the non-dominant hemisphere in contrast to the common assumption otherwise.

The explanation for post stroke auditory hallucination can be based on the development models of this phenomenon. Two main theories of the etiology of hallucinations are presently under discussion: the 'stimulation theory' and the 'inhibition theory'. The stimulation theory explains the origin of auditory hallucination as being caused by seizures or electrical and neurochemical stimulators. The inhibition theory is based on destruction of normally inhibitory function, resulting in a disinhibition phenomenon [5] that involves a reduced corticocortical connectivity neural network. Brasic [5] provided a model of auditory hallucinations by showing that excessively pruned attractor networks become functionally fragmented, and thatpruning back-propagation stimulation of speech perception leads to spontaneous output. Using neuroimaging methods, Levitan et al. [19] showed that the severity of auditory hallucinations was significantly associated with smaller left anterior superior gyrus volume, thereby demonstrating a deficit of corticocortical connection tissue. Rajarethinam et al. [22] discovered similar findings in schizophrenic patients having auditory hallucinations and proposed that these anatomical abnormalities and dysfunction of primary auditory cortex may play a role in production of this perceptual disorder.

Since all presented patients resemble those having pathological inner speech, an explanation based on the mechanism of pathological thinking in words can also be discussed. One theory of of auditory hallucination is based on the model of pathological endogenous language production process of perception leading to creation of inner speech [8]. It is postulated that the unusual phenomenon of thinking in words is a result of the brain tissue destructive process of the inner speech monitory center associated with the frontal generating area [20].

The infarcted areas in all of our patients involve overlapping brain locations, which were found to be involved in patients with auditory hallucinations in several neuroimaging studies [6,9,27,29]. Most functional imaging studies have concentrated on psychotic schizophrenic patients. Using fMRI, Dierks et al. [19] found an increase of blood oxygen level dependent signal in the Heschel's gyrus during patients' auditory hallucinations. Change in the cerebral blood flow and metabolism was also shown in the superior temporal gyrus and in the cingulate gyrus, using PET and SPECT techniques [27]. However, in rare cases of patients suffering from auditory hallucinations caused by stroke, hypoperfusion was found in the temporal lobe, indicating a decrease of blood flow in those areas [9,29].

The data from this studyreveal that auditory hallucinations can be isolated sequelae of strokes located in the dominant temporal lobe. This phenomenon seems to be more frequent than suspected and must be taken into consideration when handling patients with acute onset of auditory hallucinations.

## 6. Conclusion

This prospective study demonstrated an uncommon post stroke manifestation of auditory hallucinations (>1%). This form of perceptual disturbance cannot be explained by psychiatric or epileptogenic means, but is a consequence of vascular tissue damage or tissue dysfunction and should be taken into consideration in the clinical evaluation of brain infarction involving the temporal lobe.

## Acknowledgment

We wish to extend our appreciation to Ms. Judy Brandt for her skillful English editing and word processing expertise and contributions.
